# The Treatment of Vesico-Vaginal Fistula

**Published:** 1894-06

**Authors:** 


THE TREATMENT OF VESICO-VAGINAL FISTULA.—Augustus P. Clarke read a paper upon the above subject at the recent meeting of the American Medical Association. After considering the pathology of the condition and referring to different methods of treatment, he says: After splitting up the septum around the margin of the fistula, the deeper or vesical portions of the tissue should be brought together by means of a medium-sized tendon suture. This should be made after the manner of the cord-wainers' stitch. After the layer of sutures has been taken through the vaginal or external tissue of the bladder, the first line of sutures will become entirely concealed.

The sutures employed for the vaginal wall should be somewhat smaller than those for the vesical layer, and should be executed by introducing the needle first into one lip of the wound some distance from within and by pressing outward close to the edges of the coapted parts. After the stitches have become secured, the tissues through which they pass will superimpose and will protect them from the action of the fluids of the vaginal wall. By means of tampons of cotton or iodoform gauze to the vulvo-vaginal introitus the tissues can be kept aseptic. Catheterization of the bladder at regular intervals will prevent any undue strain on the structures of the bladder, and will help produce a speedy and permanent cure. The author has found that aseptic animal sutures are the best for this kind of work, and he prefers kangaroo tendons prepared after the formula of Lister. Aseptic catgut sutures are also very satisfactory in operations for vesico-vaginal fistula.—*Ex.*

